# Fetoplacental Weight Relationship in Normal Pregnancy and Pregnancy Complicated by Pregnancy-Induced Hypertension and Abruption of Placenta among Mothers Who Gave Birth in Southern Ethiopia, 2018

**DOI:** 10.1155/2020/6839416

**Published:** 2020-01-27

**Authors:** Tsegaye Mehare, Daniel Kebede

**Affiliations:** ^1^Department of Human Anatomy, College of Medicine and Health Sciences, Dilla University, Dilla, Ethiopia; ^2^Department of Midwifery, College of Medicine and Health Sciences, Dilla University, Dilla, Ethiopia

## Abstract

**Introduction:**

Placenta is a complex multifunctional organ that maintains pregnancy and promotes normal fetal development. The fetal outcome is adversely influenced by pathological changes in the placenta because it is a mirror that reflects the intrauterine status of the fetus. Placental abnormalities are considered a leading cause of maternal and prenatal mortality. This study aimed to assess the fetoplacental weight relationship in pregnancy-induced hypertension and abruption placenta and compare with the normal one.

**Objective:**

This study designed to assess fetoplacental weight relationships in normal pregnancy and pregnancy complicated by pregnancy-induced hypertension and abruption of placenta among mothers who gave birth in Dilla University Referral Hospital, southern Ethiopia, 2018.

**Materials and Methods:**

Institution-based comparative cross-sectional study was used on 50 placentas from mothers with pregnancy-induced hypertension, 50 placentas from mothers with abruption of placenta, and 50 placentas from mothers with normal pregnancy (control) with an age range of 19–34 years. The weight of the placenta and newborn were taken and the fetoplacental ratio was calculated.

**Results:**

Placental index as well as the weight of the newborn shows statistically significant (*p* < 0.001) difference in pregnancy-induced hypertension and abruption placenta group compared with the normal group. The mean of the fetoplacental ratio in the normal group was 5.52 ± 0.07, in pregnancy-induced hypertension was 5.15 ± 0.11, whereas the abruption placenta was 4.99 ± 0.82.

**Conclusion:**

Both PIH and abruption placenta were associated with remarkable changes in the placenta index such as small placental weight and diameter and results in different kinds of congenital anomalies and low birth weight of the baby. Hence, fetoplacental ratio was altered. The lowest fetoplacental ratio was 4.99 for abruption placenta, and the highest was for a normal group of the placenta which was 5.52. Therefore, an examination of the placenta before and after birth guarantees for feto-maternal health.

## 1. Introduction

The placenta is a complex multifunctional organ that maintains pregnancy and promotes normal fetal development [1]. The fetal outcome is adversely influenced by pathological changes observed in placenta because it is a mirror that reflects the intrauterine status of the fetus [2]. Placental anomalies are the leading cause of maternal and prenatal mortality and important factors affecting fetal growth. Several studies have been conducted in developed countries which have suggested that placental indices have a significant role in fetal growth in terms of weight and congenital anomalies [3, 4].

Hence, a thorough examination of the placenta in the uterus and during parturition morphometrically provides much insight into the prenatal health of the baby and the mother [5–7]. But currently, very little is known about the incidence of fetal deaths resulting from placenta malformation in most developing countries including our country [8–10]. Fetal outcome risks due to the different levels of maturation are also still unknown in our country [8, 10]. Therefore, the present study is designed to provide baseline information on the measurement of placenta along with fetal weight and calculate the fetoplacental ratio (FTP) in pregnancy-induced hypertension (PIH) as well as abruption placenta group and compare with FTP ratio in the normal group. So, this study aimed to measure placental weight, diameter, and fetal weight and after that FTP ratio was calculated in PIH plus abruption placenta (APH) group and compared with placental and fetal index in the normal placental group.

## 2. Methods and Materials

The institution-based comparative cross-sectional study design was used. Informed assent was not taken from the mother of each study participants (neonates) because measuring weight and performing a physical examination for the newborn was part of daily practice in the gynecology and obstetrics ward. Placenta discarded as a waste, so no ethical approval is required. The research involved has no risk to subjects. A study began with the identification of the cases. 50 placentas collected from parturient having blood pressure ranged 140/90 mm of Hg or above were grouped as PIH group.

50 placentas from parturient with a history of abruption placenta were considered as abruption placental group and 50 placentas from parturient free from any pregnancy complications (diseases) belonged to the normal group (control group). Data collected starting December to July 2018 from the labor room and gynecology operation theatre at Dilla University Referral Hospital.

### 2.1. Steps for Data Collection

- Maternal clinical history was a review from the card
- Freshly delivered placentas were collected from the labor and gynecology operation theatre
- Washed with normal saline and measured its diameter and weight
- After careful observation, the picture was taken using a 21-megapixel Canon camera
- Fetal weight was measured using a standard weight scale

### 2.2. Data Quality Control

Quality of the data would be assured by a properly designed check list, and each placental as well as fetal index was measured by two different occasions via two examiners, and the results obtained were compared and ratified. Each day after data collection, the check list was reviewed and checked for completeness and relevance by the supervisor and principal investigator and the necessary feedback had been offered to the data collectors in the next morning.

### 2.3. Data Processing and Analysis

SPSS version 23 was used for data entry, cleaning, and analysis. Independent *t*-test and Dunnett C test was used to find the significant difference between mean weight and diameter of the placenta from the PIH plus abruption placenta group to the normal placenta group. Univariate analyses were performed for “Dunnett's C” and Games-Howell test to find a statistically significant difference in mean of FTP ratio among PIH placental group vs. normal group and abruption placental group vs. normal group. Mean difference was expressed at 95% CI as appropriate. *p* < 0.05 was considered significant for all cases.

## 3. Results

### 3.1. Sociodemographic Characteristics of Participants

A total of 150 (50 PIH, 50 abruptions, and 50 normal) participants were involved in this study with a response rate of 100%. The mean ages of the mothers of PIH, abruption history, and normal were 27.12 (SD = 3.84), 27.02 (SD = 3.97), and 26.74 (SD = 4.3) years, respectively. 60% (30) of the respondents having PIH and abruption placenta were in the age group between 23 and 30 years, but in the case of normal women, age was almost uniform throughout each age group (Table 1). The primiparous was 34% in all the groups. Gravid 1-2 were equal in normal and abruption placenta groups and representing 30% for each group, whereas 17 cases/34% were seen in PIH. Gravid 3–7 were 38% for the abruption placenta group, while they were 32% for the PIH group and 36% in the normal group (Table 1).

### 3.2. Placental-Related Parameters

**Figure 1:**
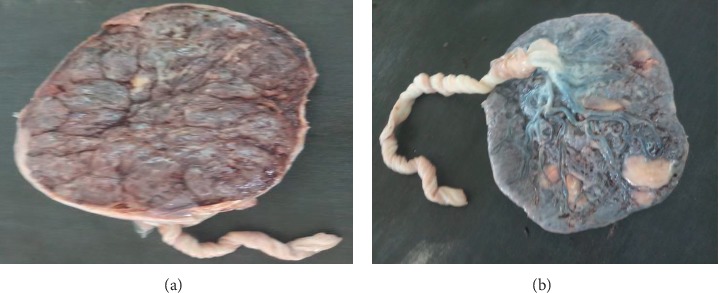
Gross morphology of normal placenta on maternal and fetal surfaces, respectively.

**Figure 2:**
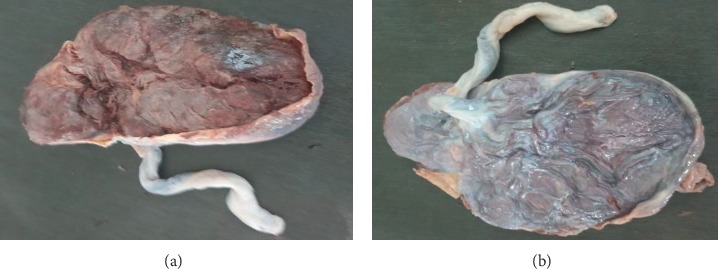
Gross morphology of placenta in pregnancy-induced hypertension on maternal and fetal surfaces, respectively.

**Figure 3:**
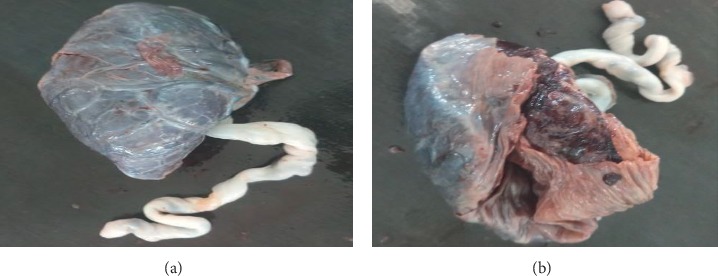
Gross morphology of abruption placenta on maternal and fetal surfaces, respectively.

The weight of 50 placentas in the normal group ranges from 400 gm to 625 gm with a mean weight of 524.36 ± 8.1 gm (mean ± SEM) (Table 2, Figure 1). In 50 placentas of the PIH group, the weight ranges from 280 gm to 611 gm with a mean weight of 461.36 ± 12.64 gm (mean ± SEM) (Table 2, Figure 2). In 50 cases of abruption placenta, the weight ranges from 280 gm to 589 gm with a mean weight of 408.02 ± 10.92 gm (mean ± SEM) (Table 2, Figure 3).

In our study, the placental weight shows statistically significant (*p* < 0.001) difference in the PIH and abruption placenta group as compared with the normal group (Table 2).

The diameter of the placenta in the normal group ranges from 14 cm to 27 cm with a mean of 21.74 ± 0.74 cm (mean ± SEM). In the PIH group, the diameter ranges from 11 cm to 28 cm with a mean of 18.92 ± 0.72 cm (mean ± SEM), but in the abruption placenta group, the diameter ranges from 10 cm to 29 cm with a mean of 15.22 ± 0.64 cm (mean ± SEM). In our study, placental diameter shows statistically significant (*p*=0.004) difference in PIH as compared with the normal group and also abruption placenta group compared with the normal group (*p* < 0.001) (Table 2).

### 3.3. Neonatal-Related Parameters

The mean weight of the newborn from mothers of PIH and abruption placenta group compared with the normal group shows a significant difference (*p* < 0.001) on statistical analysis (Table 3).

The mean of FTP ratio in the normal group was 5.52 ± 0.07 (mean ± SEM), in PIH group was 5.15 ± 0.11 (mean ± SEM), whereas in abruption placenta group, it was 4.99 ± 0.82 (mean ± SEM). Normal group means FTP ratio showed highly significant difference (*p* < 0.05) compared with PIH and abruption placenta group mean (Table 4).

## 4. Discussion

The placenta is a complex multifunctional organ of mainly fetal origin with pleiotropic roles during fetal growth [1]. It is a mirror that reflects the intrauterine status of the fetus [11]. Placenta maintains pregnancy and promotes normal fetal development as well as a window that provides insight vision for understanding maternal dysfunction and its impacts on fetal health [8]. Through an examination of the placenta both at the pre- and postnatal periods is very important because any kind of anomalies on this critical organ causes many fetal abnormalities. The placental examination gives valuable information about the state of the fetal wellbeing [5, 6]. Especially in a community like ours where most antenatal mothers still come unbooked to the labor room with no prior investigations done, and midwives and gynecologist should not ignore to carry out a placental examination.

In the current study, the mean weight of placenta decrease in PIH and abruption placenta than the normal placenta. Highly statistical significant (*p* < 0.001) difference was seen in the weight of the placenta for PIH and abruption placenta group as compared with the normal group (Table 2, Figures 1–3). The mean placental weight difference was 63 gm at 95%, CI: 27.18–98.82 between the normal group and PIH group whereas between normal and abruption placenta groups the mean placental weight difference was 116.34 gm at 95% CI 83.95–148.73 (Table 5). A similar study done by Pushpa Goswami et al. in 2013 reported less placental diameter in (66%) of cases in patients of PIH and abruption placenta [12]. According to Palaskar et al. reduced placental weight in 77% [13].

In this study, mean placental diameter shows a highly significant (*p* < 0.001) difference in PIH and abruption placenta group as compared with the normal group (Table 2, Figures 1–3). The mean placental diameter difference between normal and PIH group was 2.28 at 95% CI 0.76–4.88, whereas between normal and abruption placenta groups, the mean placental diameter difference was 6.52 at 95% CI 4.62–8.4 (Table 4). Sultana et al. in 2007 have reported less placental diameter in (55%) of cases in patients of PIH [14], whereas different results have been observed by Ashfaq et al. in 2005 showing no difference in weight and diameter in the placenta with PIH and normal groups [15]. However, a difference in study designs, data collection methods, and controlling for different factors make a comparison of the results difficult.

In this study and a study performed by Udaina and Jain in 2001 [11] and Sarwar and Islam in 2006 uteroplacental insufficiency is found to be the leading cause of low birth weight and other congenital anomalies for neonates [16]. The weight of a newborn in normal placental group ranges from 1.8 kg to 3.5 kg with a mean weight of 2.9 ± 0.06 kg (mean ± SEM), and in abruption placenta group, fetal weight ranges from 1.3 kg to 3.6 kg with a mean weight of 2.07 ± 0.96 kg (mean ± SEM), whereas in the PIH group, the fetal weight ranges from 1.4 kg to 3.4 kg with a mean weight of 2.41 ± 0.1 kg (mean ± SEM) (Table 3). The newborn weight reduction was mainly noted for the abruption placenta group but also slightly noted in the PIH group. The weight shows a highly significant (*p* < 0.001) difference in the abruption placenta and PIH group as compared with the normal group. In this study, the mean newborn weight difference between normal and abruption placenta group was 0.8254 at 95% CI 0.0.5587–1.092, whereas between normal and PIH groups, the mean weight difference was 0.49 at 95%, CI: 0.2144–0.7656 (Table 6). The current study did not confirm the results of Rahman et al. which shows that pregnancy-induced hypertension was found to be an independent risk factor for low birth weight [17]. However, the difference in the values to some extent may be due to differences in study designs, data collection methods, ethnicity, and controlling for confounders.

During pregnancy, the placental mass maintains a dynamic relationship with the weight of developing a fetus. In this study, mean of FTP ratio in a normal group was 5.52, in PIH, it was 5.15, whereas in the abruption of placenta, it was 4.99 (Table 7). When the normal group was compared with PIH and abruption placenta on the Dunnett C test, it shows a highly significant difference.

Decreased FTP ratio noted for the abruption placenta group but almost the same to normal and PIH groups when we compare with other similar studies. Morphologically placenta of hypertensive disorders of pregnancy are lighter in weight, lesser in diameter, thickness, and the fetoplacental ratio is diminished because the rate of reduction of baby weight was less than that of the rate of reduction of placental weight in PIH as in such disease conditions placenta worked extensively for its function with limited tissue.

Pushpa Goswami et al. observed the fetoplacental ratio in the normal group is 5.38, in the PIH group, it was 5.097, whereas in the abruption of placenta, it is 6.7. According to Pushpa Goswami et al., placental insufficiency was associated with preterm birth, neonatal morbidity, and altered placental dimensions [18]. Palaskar observed mean fetoplacental ratio in normal pregnancy was 5.8 and 7 : 1, and in PIH, the mean fetoplacental ratio was increased to 6.04. While Gunapriya in 2011 observed the fetoplacental ratio of 5.35 in normal and in PIH, 6.03 [19], Ananth observed in abruption of placenta mean birth weight and placental weight were lower especially in preterm births with placental ratio <10^th^ centile risk ratio 0.4, 95% CI 0.2–0.8 [20]. Several studies show reduced placental weight in abruption also a low birth weight of newborn, but the fetoplacental ratio was not calculated which was significant in our study. More or less our findings are parallel with the studies conducted in the past except for the FTP ratio in the case of abruption placenta which is very low as compared with another study result. The difference in the values to some extent may be due to ethnicity, economic difference, and diet especially mothers who are a strict vegetarian.

## 5. Conclusion

Both PIH and abruption placenta was associated with remarkable changes in the placenta index such as small placental weight and diameter and results in different kinds of congenital anomalies, and low birth weight for the baby which also altered the fetoplacental ratio. The lowest fetoplacental ratio was 4.99 for abruption placenta, and the highest was the normal group of the placenta which was 5.52. Abruption placenta and PIH negatively affected both fetal and placental outcomes.

## Figures and Tables

**Table 1:** Age groups and parity of parturient women who gave birth in Dilla University Referral Hospital, southern Ethiopia.

	Normal	PIH	Abruption placenta
*Age groups*			
19–22 years	12 (24%)	7 (14%)	9 (18%)
23–26 years	14 (28%)	20 (40%)	15 (30%)
27–30 years	11 (22%)	10 (20%)	15 (30%)
31–34 years	13 (26%)	13 (26%)	11 (22%)

*Parity*			
P0	17 (34%)	17 (34%)	16 (32%)
P1-2	15 (30%)	17 (34%)	15 (30%)
P3–7	18 (36%)	16 (32%)	19 (38%)

**Table 2:** Comparative results of placental coefficient of normal (control), PIH, and abruption placenta groups collected from pregnant women who gave birth in Dilla University Referral Hospital, southern Ethiopia.

	Min. wt (gm)	Max. wt (gm)	Mean (cm)	Std	SEM	*p* value
*Placental weight in*						
Normal	400	625	524.36	57.27	8.10	
PIH	280	611	461.36	89.35	12.64	<0.001
Abruption placenta	280	589	408.02	77.19	10.92	<0.001

*Placental diameter*						
Normal	14	27	21.74	3.33	0.47	
PIH	11	28	18.92	5.11	0.72	0.004
Abruption placenta	10	29	15.22	4.53	0.64	<0.001

**Table 3:** Comparative results of newborn weight coefficient of normal (control), PIH, and abruption placenta groups collected from pregnant women who gave birth in Dilla University Referral Hospital, southern Ethiopia.

Weight of a newborn in (kg)	Min. wt (kg)	Max. wt (kg)	Mean (kg)	Std	SEM	*p* value
Normal	1.8	3.5	2.90	0.408	0.06	
PIH	1.4	3.4	2.41	0.706	0.10	<0.001
Abruption placenta	1.3	3.6	2.07	0.676	0.96	<0.001
Feto-placental ratio						
Normal	4.4	6.5	5.52	0.51	0.07	
PIH	3.8	6.4	5.15	0.79	0.11	0.016
Abruption placenta	3.7	7	4.99	0.82	0.12	0.001

**Table 4:** Placental diameter statistics in “Dunnett's C” multiple comparison tests.

Dunnett's C multiple comparison test	Mean difference (cm)	*q*	Sig (*p* < 0.05)	95% CI of difference
Normal vs. PIH	2.82	0.863	Yes	0.73–4.91
Normal vs. APH	6.52	0.795	Yes	4.60–8.44

**Table 5:** Placental weight statistics in “Dunnett's C” multiple comparison tests.

Dunnett's C multiple comparison tests	Mean difference (gm)	*q*	Sig (*p* < 0.05)	95% CI of difference
Normal vs. PIH	63.0	15.0	Yes	26.72–99.28
Normal vs. APH	116.3	13.6	Yes	83.49–149.19

**Table 6:** Newborn weight statistics in “Dunnett's C” multiple comparison tests.

Dunnett's C multiple comparison test	Mean difference (kg)	*q*	Sig (*p* < 0.05)	95% CI of difference
Normal vs. PIH	0.49	0.1154	Yes	0.2112–0.7688
Normal vs. APH	0.83	0.1117	Yes	0.5554–1.0953

**Table 7:** Fetoplacental ratio statistics in “Dunnett's C” multiple comparison tests.

Dunnett's C multiple comparison test	Mean difference	*q*	Sig (*p* < 0.05)	95% CI of difference
Normal vs. PIH	0.3760	0.1328	Yes	0.55–0.697
Normal vs. APH	0.5340	0.1362	Yes	0.205–0.863

## Data Availability

The dataset used to support the findings of this study was supplied by Tsegaye Mehare under license and so cannot be made freely available. Requests for access to these data should be made to Tsegaye Mehare, tseyeshe96@gmail.com.
